# Focal Adhesion Kinase-Dependent Role of the Soluble Form of Neurotensin Receptor-3/Sortilin in Colorectal Cancer Cell Dissociation

**DOI:** 10.3390/ijms17111860

**Published:** 2016-11-08

**Authors:** Sophie Béraud-Dufour, Christelle Devader, Fabienne Massa, Morgane Roulot, Thierry Coppola, Jean Mazella

**Affiliations:** Centre National de la Recherche Scientifique, Institut de Pharmacologie Moléculaire et Cellulaire, UMR 7275, Université Côte d’Azur, 660 route des Lucioles, 06560 Valbonne, France; beraud@ipmc.cnrs.fr (S.B.-D.); devader@ipmc.cnrs.fr (C.D.); fabiennemassa@gmail.com (F.M.); roulot@ipmc.cnrs.fr (M.R.); coppola@ipmc.cnrs.fr (T.C.)

**Keywords:** soluble neurotensin receptor-3, sortilin, neurotensin, cancer, cell signaling, cell morphology

## Abstract

The aim of the present review is to unravel the mechanisms of action of the soluble form of the neurotensin (NT) receptor-3 (NTSR3), also called Sortilin, in numerous physiopathological processes including cancer development, cardiovascular diseases and depression. Sortilin/NTSR3 is a transmembrane protein thought to exert multiple functions both intracellularly and at the level of the plasma membrane. The Sortilin/NTSR3 extracellular domain is released by shedding from all the cells expressing the protein. Although the existence of the soluble form of Sortilin/NTSR3 (sSortilin/NTSR3) has been evidenced for more than 10 years, the studies focusing on the role of this soluble protein at the mechanistic level remain rare. Numerous cancer cells, including colonic cancer cells, express the receptor family of neurotensin (NT), and particularly Sortilin/NTSR3. This review aims to summarize the functional role of sSortilin/NTSR3 characterized in the colonic cancer cell line HT29. This includes mechanisms involving signaling cascades through focal adhesion kinase (FAK), a key pathway leading to the weakening of cell–cell and cell–extracellular matrix adhesions, a series of events which could be responsible for cancer metastasis. Finally, some future approaches targeting the release of sNTSR3 through the inhibition of matrix metalloproteases (MMPs) are suggested.

## 1. Introduction

Cancer cell development is under the control of the abnormal expression of growth factors whereas cancer cell metastasis is the consequence of the weakening of cell-cell interactions leading to the release of tumor cells in the blood circulation [1]. Both cancer growth and dissemination are activated by numerous extracellular activators including neuropeptides [2] and factors released by shedding from the plasma membrane which depends on matrix metalloproteases (MMPs) such as Epidermal Growth Factor Receptor (EGFR) ligands [3]. The transmembrane protein members of the Vps10p protein family [4,5] are also shedded by similar mechanisms that release the extracellular part of Sortilin/NTSR3, SorLa and sortilin-related receptors expressed in the central nervous system (SorCs1–4) proteins [6,7]. However, except for Sortilin/NTSR3, their involvement in cancer proliferation has not been clearly demonstrated. The type I transmembrane protein Sortilin [8], also identified as neurotensin (NT) receptor-3 (NTSR3) [9], is a multifunctional protein regulating numerous intracellular and tissue functions such as the sorting of proteins to lysosomes [10] or to the plasma membrane [11]. Sortilin/NTSR3 also acts as a receptor for NT [12], a lipoprotein lipase [13], or a co-receptor to trigger the actions of NT [14,15], the precursor of Nerve Growth Factor (pro-NGF) [16], and also as the precursor of Brain Derived Neurotrophic Factor (pro-BDNF) [17]. Sortilin/NTSR3 has been recently identified as a receptor involved in Alzheimer’s disease [18]. In the field of cancer, the role of this protein has been initially documented by its implication in the NT-induced proliferation of several cancer cell lines [19]. Then, Sortilin/NTSR3, in association with the neurotrophin receptor tropomyosin-related kinase B (TrkB) and the 75 kDa neurotrophin receptor (p75NTR), has been shown to play a key role in the survival of B cells [20] and also as essential for colorectal cancer cell growth [21]. Interestingly, when associated with the TrkB receptor as well, Sortilin/NTSR3 is also expressed in exosomes to promote the transfer of glioblastoma aggressiveness to healthy cells [22]. Finally, immunohistochemistry experiments performed in a cohort of clinical breast cancers and normal breast tissues revealed an increase in the expression of Sortilin/NTSR3 associated with breast cancer aggressiveness, particularly in ductal invasive carcinomas and in association with lymph node invasion [23]. The observation that Sortilin/NTSR3 is shedded according to MMP-dependent mechanisms [6] would attract the interest of several laboratories in order to examine the functional relevance of the resulting soluble form (sSortilin/NTSR3). However, evidence on the role of this soluble protein remains poorly documented.

Numerous cells, including neurons as well as normal and cancer cells, express the family of neurotensin (NT) receptors, and particularly Sortilin/NTSR3 [24,25]. All these cells release the extracellular domain of Sortilin/NTSR3 from the plasma membrane after a shedding process dependent on the activation of protein kinase C (PKC). The neurotensinergic system plays an important role in the development of lung cancer in association with tyrosine kinase receptors [26,27,28]. In particular, Sortilin/NTSR3 was described to mediate the release and transfer of exosomes from the human cancer cell line A549 to endothelial cells, resulting in an increase in the phosphorylation of proteins involved in cell growth and differentiation (e.g., extracellular signal-regulated kinase Erk1/2 and Akt) [27]. However, the function of the soluble form of Sortilin/NTSR3 was not investigated in this type of cancer. Therefore, this review summarizes the functional role of sSortilin/NTSR3 essentially on colonic cancer cells through mechanisms involving signaling pathways leading to cell–cell weakening and cell–extracellular matrix loss of adhesion. These events could be crucial for cancer cell dissemination and metastasis. Future approaches targeting the release of sSortilin/NTSR3 by inhibition of MMPs are discussed.

## 2. Release, Binding and Internalization Properties of sSortilin/NTSR3

### 2.1. Shedding

**Figure 1:**
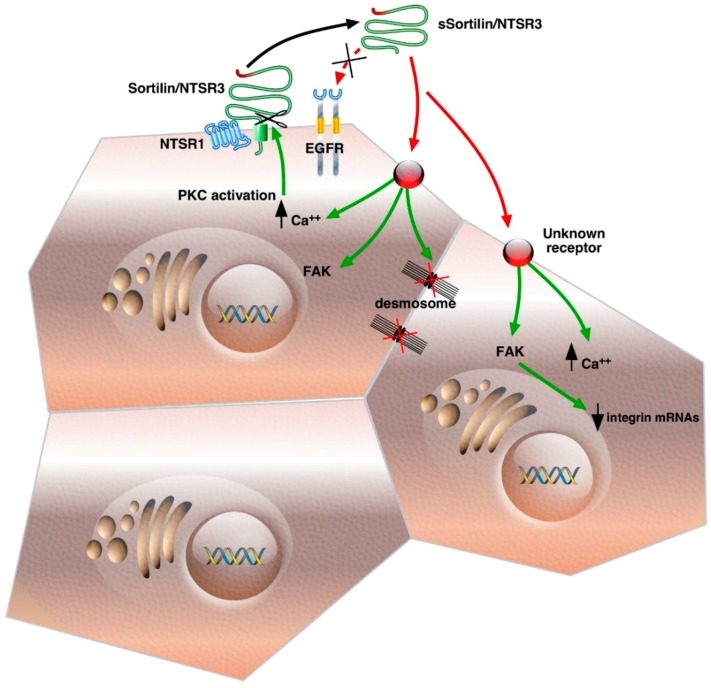
Schematic representation of Sortilin/NTSR3 shedding and signaling cascades in HT29 cells; green, intracellular pathways and red, extracellular interactions. The abbreviations used are: Ca^++^, intracellular calcium; NTSR1, Neurotensin Receptor-1; Sortilin/NTSR3, Neurotensin Receptor-3/Sortilin; EGFR, Epidermal Growth Factor Receptor; PKC, Protein Kinase C; FAK, Focal Adhesion Kinase. Green arrows, intracellular activated pathways; Red arrows, extracellular interactions; Up and down black arrows, increased or decreased levels.

Sortilin/NTSR3 was first shown to be released from several types of cells such as neurons, microglial cells and cancer cells as a protein with a molecular weight of 100 kDa, slightly lower than the one detected in crude homogenates (110 kDa) [6]. The shedding of Sortilin/NTSR3 was not activated by NT in the HT29 cell line but the amount of sSortilin/NTSR3 recovered in the extracellular medium was enhanced when the internalization process was blocked by hyperosmolar sucrose, suggesting an accumulation of the protein at the cell surface and also an increase of the amount of shedded protein in these conditions. Interestingly, this shedding process is activated in a concentration- and time-dependent manner by PMA (Phorbol 12-Myristate 13-Acetate), an activator of MMPs via the PKC pathway (Figure 1). In the same way, other PKC activators such as carbachol or Prostaglandin E2 (PGE2) [29] are able to increase the shedding of Sortilin/NTSR3 [30]. Other members of the Vps10p receptor family, SorLA and SorCS1-3, are not only shedded [7,31], but they are also gamma-secretase substrates [32], suggesting that both extracellular and intracellular released domains of the Vps10p receptor family may have functional activities in neurodegenerative diseases as described for SorLA in the pathology of Alzheimer’s disease [33].

### 2.2. Binding of sSortilin/NTSR3 to a Specific Receptor Independent on the Epidermal Growth Factor Receptor (EGFR)

The soluble forms of Vps10p proteins could display their own activities as ligands or could serve, by their ability to bind their extracellular ligands, as transporters/protectors to avoid their proteolytic degradation. In the case of Sortilin/NTSR3, binding experiments performed on HT29 cell homogenates using radiolabeled protein indicate that sSortilin/NTSR3 is a ligand able to specifically bind to HT29 membranes with an affinity of 5 nM, but not on the NT receptors [30]. From the fact that sSortilin/NTSR3 is released by mechanisms similar to those leading to EGFR ligands and that in numerous cancer cell systems NT signaling depends on EGFR activation [34,35], the verification that sSortilin/NTSR3 and EGF could be competitive ligands has been made. In summary, sSortilin/NTSR3 is unable to compete with EGF on EGFR, and reciprocally, EGF is unable to compete with sSortilin/NTSR3 on its binding sites [30]. The absence of the sSortilin/NTSR3 interaction with the EGFR system is confirmed by the absence of the effect of the soluble protein both on Erk1/2 signaling and cell growth induced by EGF. All together, these observations indicate that sSortilin/NTSR3 recognizes a specific receptor in HT29 cells that is neither sortilin nor EGFR since NT does not compete with its binding sites (Figure 1).

### 2.3. Internalization Properties of Sortilin/NTSR3

Interestingly, internalization experiments performed with either radioiodinated or fluorescent sSortilin/NTSR3 demonstrated that about 40% of the total bound soluble protein was rapidly and efficiently sequestrated at 37 °C into HT29 cells by a mechanism dependent on hyperosmolar sucrose [30]. Following its internalization, part of sSortilin/NTSR3 was recovered into Lysotracker-labeled lysosomes where 60%–70% of the sequestered protein was degraded after 45 min. The internalization of sSortilin/NTSR3 protein that was partly processed to lysosomes likely indicated that the membrane receptor protein that recognized sSortilin/NTSR3 could be regulated by a clathrin-dependent mechanism. The remaining intact intracellular sSortilin/NTSR3 could be sorted to recycling vesicles or to other cellular compartments to trigger unidentified functions. Intriguingly, although it was demonstrated that Sortilin/NTSR3 was not the receptor of sSortilin/NTSR3, both the soluble and the membrane-bound proteins seem to follow the same sorting to lysosomes [36].

## 3. Signaling of sSortilin/NTSR3 in HT29 Cells

### 3.1. Calcium and Protein Kinase C (PKCα) Translocation

The functionality of sSortilin/NTSR3 was first demonstrated by its ability to increase the intracellular concentration of calcium at a lower concentration of 10 nM, close to its affinity for its binding sites. sSortilin/NTSR3 also induces plasma membrane translocation of the PKCα, suggesting that the effect of the soluble protein on the calcium concentration is the consequence of PKCα activation [30]. Repeated incubations with sSortilin/NTSR3 lead to a decrease in calcium concentration as a result of the desensitization of the system, a phenomenon frequently demonstrated by the internalization and uncoupling of functional receptors such as G-protein coupled receptors [37] and the low-density lipoprotein lipase receptor family [38]. This result argues for the existence of a functional specific receptor that can be desensitized.

### 3.2. Focal Adhesion Kinase (FAK) Dependent Stimulation of the PI3 Kinase Pathway

The phosphatidylinositol 3-kinase (PI3 Kinase) pathway activation visualized by the phosphorylation of Akt, for example, is an important step to induce calcium release from the intracellular stores (for review, see [39]), a pathway involved in the development of colorectal cancers [40]. In HT29 cells, low concentrations of sSortilin/NTSR3 (10 nM) rapidly and transiently enhanced Akt phosphorylation through the upstream phosphorylation of the complex FAK–Src. This was demonstrated by the use of the FAK inhibitor II that totally inhibited the effect of sSortilin/NTSR3 on Akt phosphorylation [30]. These findings are crucial since the activation of the FAK pathway is described to be involved in survival mechanisms, and especially in a variety of distinct cancer cell lines’ development and metastasis [41,42]. Indeed, FAK regulates numerous downstream intracellular pathways that mediate either cell migration through actin modification [43] or cell proliferation through transcription factors [44]. Interestingly, in HT29 cells, both the actin cytoskeleton and mitogen-activatd protein (MAP) kinases are regulated by sSortilin/NTSR3.

## 4. Morphological Changes of HT29 Cells Induced by sSortilin/NTSR3

Since the activation of the FAK pathway is strongly correlated to numerous cellular processes such as cell spreading, adhesion, migration and survival [45], studying the HT29 cell shape and morphology upon sSortilin/NTSR3 incubation added important answers about the role of the protein in the regulation of cancer cell detachment [46].

### Cell Shape and Size, Cytoskeleton and Cell–Cell Junctions Modification

The geometric distribution (polygon classes) of cells, a property known to reflect their modifications of states [47], was assessed using labeling with E-cadherin antibodies to visualize cell outlines by confocal imaging. Resting confluent HT29 cells presented a geometric distribution corresponding to 46% of hexagons as shown by the schematic representation in Figure 1, a distribution consistent with several other resting cells [47,48]. Interestingly, HT29 cells treated with sSortilin/NTSR3 displayed not only a significant reduction (to 30%) in the proportion of hexagons in favor of pentagons, but also an increase in the cell surface [46]. However, additional studies dealing with the determination of the effect of sSortilin/NTSR3 on cell volume are still missing.

The modifications of the cell shape upon sSortilin/NTSR3 treatment were consistent with a reorganization of the actin cytoskeleton characterized by the increase of actin stress fibers and the disruption of actin microfilaments surrounding the inner side of the membranes of peripheral cells. The modifications of the actin cytoskeleton as well as the ability of sSortilin/NTSR3 to activate FAK were certainly linked to the cell-matrix contact weakening, which can lead to cell migration. However, since HT29 cells are non-migrating cells [49], the role of sSortilin/NTSR3 likely corresponds to an involvement in the first step of a mechanism responsible of cell detachment.

## 5. Cell–Cell and Cell–Matrix Junctions

### 5.1. Desmosomes Disruption

The reorganization of actin microfilaments and the change of the HT29 cell shape induced by sSortilin/NTSR3 are in agreement with the modifications observed in the architecture of some ultrastructural components, particularly desmosomes and intermediate filaments [46]. Desmosomes are formed of plaque densities and bundles of intermediate filaments. These structures participate in the fortification of cell–cell adhesion by connecting the proteins forming plaque densities to the interfilaments’ cytoskeleton. Desmosomes are crucial to keep tissue integrity and homeostasis [50]. In HT29 cells treated with sSortilin/NTSR3, the average number of desmosomes per cell dramatically decreased, and moreover, the architecture of desmosomes was modified. Indeed, the most important perturbation observed upon sSortilin/NTSR3 treatment was the loss of the association of intermediate filament bundles with the plaque densities. This results in the disruption of the desmosomes’ integrity (Figure 1), an additional property of sSortilin/NTSR3 in the embrittlement of cell–cell and cell–matrix interaction. The desmosomes’ integrity is crucial to maintain cell cohesion, and its structure disorganization may be responsible for the weakening of the cell barrier that can allow the crossing of growth factors leading to many tissue dysfunctions, and particularly in the development or the progression of human epithelial cancer cells (for reviews, see [51,52]). 

### 5.2. Modification of Cadherin and Integrins

Briefly, cadherins participate in cell-cell adhesion through intercellular structures called adherens junctions (for review, see [53]). Integrins are transmembrane proteins involved in the adhesion to the extracellular matrix [54]. Integrins are correlated to the FAK/Src complex that modulates small GTPases and then actin cytoskeleton remodeling [55].

The marked changes observed in the sSortilin/NTSR3-treated HT29 cell morphology are correlated with the modifications of the expression of proteins implicated in cell-cell junctions or cell adhesion. The determination of the mRNA expression of E-cadherin and integrins by quantitative PCR revealed that a 6 h treatment with sSortilin/NTSR3 is enough to almost totally decrease the expression of E-cadherin. The expression of several integrins, including α1, α7, αV, β4, β6 and β8, was also reduced upon sSortilin/NTSR3 treatment [46]. The decrease of E-cadherin expression likely corresponds to a more diffuse labeling of the protein observed at the cell–cell junction which probably leads to the weakening of cell–cell cohesion. The sSortilin/NTSR3-induced inhibition of a number of integrin family members could be responsible for the cell–matrix detachment by decreasing the capacity of the cell to maintain solid contacts with the matrix. All together, these properties show that sSortilin/NTSR3 may be involved in the first steps leading to cancer cell detachment from a primary tumor, a process that induces cell dissemination and then metastasis. Decrease or loss of integrin couples has been described in lung adenocarcinoma [56,57] and in colonic epithelial cells [58,59] in association with a poor prognosis.

### 5.3. Cancer Cell Detachment

In agreement with previous observations on the decrease of the expression of E-cadherin and on several integrins adding to cell morphology changes after sSortilin/NTSR3 treatment, the weakening of cell–cell contact and cell-matrix interaction led to cell detachment from the plates. This was measured in several colonic cancer cells including HT29, HCT116 and SW620 cell lines. Taken together, all the properties characterized in cancer cells may reflect a potent action of sSortilin/NTSR3 on cell attachment weakening leading to dissemination of cancer cells (Figure 1).

## 6. Other Functions of sSortilin/NTSR3

The amount of sSortilin/NTSR3 present in the blood circulation could be a bad or a good predictor for several pathologies including, for example, cardiovascular diseases or depression. Its ability to bind numerous circulating peptides and proteins would allow researchers to develop new concepts for treatments of these pathologies by targeting sSortilin/NTSR3.

### Atherosclerosis and Depression

Indeed, a very recent work reported that the plasma level of sSortilin/NTSR3 was increased by activated platelets in association with cardiovascular risk from patients with hypertension and dyslipidemia [60]. Interestingly, statin treatment of patients with cardiovascular risks enhanced the amount of plasma Proprotein Convertase Subtilisin/kexin type 9 (PCSK9), a convertase responsible for the degradation of low-density lipoprotein (LDL) receptors [61]. The increase in the plasma level of PCSK9 was correlated with the decrease of sSortilin/NTSR3 [62]. Since sSortilin/NTSR3 decreases the level of LDL through the internalization process [63], its decreased plasma level under statin treatment may counteract the increase of PCSK9 that is deleterious for the LDL level.

Soluble Sortilin/NTSR3 has also been detected in cerebrospinal fluid and its concentration increases with age and is positively correlated with progranulin [64]. Since the full-length Sortilin/NTSR3 binds progranulin, followed by its endocytosis and lysosomal degradation [65], the soluble protein also binds with a high affinity to progranulin [65] and may compete with the membrane-bound receptor to potentially protect progranulin from degradation.

Finally, increased serum concentrations of sSortilin/NTSR3 have been detected in patients suffering from major depression when compared to control healthy subjects [66]. Interestingly, the higher level of sSortilin/NTSR3 in depressive patients is correlated with an increase of BDNF and VEGF levels, indicating that the circulating sSortilin/NTSR3 could be a new candidate as a biomarker of depression state.

However, the amount of sSortilin/NTSR3 measured in the works presented above can be skewed by the full-length membrane-bound Sortilin/NTSR3 that can be present in exosomes released from different blood cell types and in other fluids. Exosomes are small vesicles (30–100 nm in diameter) that can be released in all fluids including plasma/serum, saliva, cerebrospinal fluid, urine, etc. These vesicles contain multiple proteins, DNA, RNA, miRNA and even materials of viruses. Exosomes appear to be responsible for the dissemination of several important pathologies and particularly in cancer progression [67]. Recently, it has been demonstrated that Sortilin/NTSR3 is a key component of exosome biogenesis in association with two tyrosine kinase receptors (TrkB and EGFR) [27,68]. This has been observed in the human lung cancer cells (A549). Sortilin/NTSR3 (the membrane bound form) is then likely recovered in the majority of exosomes in the blood circulation, and such a concentration can easily modify the “apparent” amount of the soluble form.

## 7. Conclusions

Both forms of Sortilin/NTSR3, the full-length membrane-bound and the soluble extracellular domain forms, display numerous physiological and pathological functions. Their regulations involve also multiple and complex mechanisms.

The release of sSortilin/NTSR3 depends on the activation of the protein kinase C upon external stimuli, which leads to the activation of MMPs that cleave the luminal part of Sortilin/NTSR3. Once released in the medium, sSortilin/NTSR3 binds to specific binding sites to trigger numerous intracellular effects including the activation of PKCα, suggesting an autoregulation of the turnover of the soluble protein. It is important to underline that effective concentrations of sSortilin/NTSR3 that induce PKCα activation and calcium increase are around 10 nM, concentrations that are in agreement with the affinity of sSortilin/NTSR3 to its binding sites. Interestingly, these concentrations also correspond to the serum levels of sSortilin/NTSR3 determined in several works. 

The sSortilin/NTSR3 receptor is not known yet. However, one hypothesis is that the receptor is a complex constituted by the membrane-bound Sortilin/NTSR3 and some integrins to form strong links between the cells and between the cells and matrix. In this case, the sSortilin/NTSR3 can compete with the membrane-bound protein, leading to the weakening of these interactions as observed in HT29 cells. The resulting effect could finally be the dissociation and the dissemination of cancer cells responsible for metastasis. The putative complex between Sortilin/NTSR3 and integrin(s) may be also responsible of the activation of FAK as observed in HT29 cells since integrins are able to stimulate intracellular kinases including FAK-Src (for review, see [69]).

Since sSortilin/NTSR3 appears to display an important role in the development of metastasis and its seric levels are also deleterious for cardiovascular risks and depression, its regulation could be a new challenge to reduce its release by targeting the activity of MMPs. One good example is the use of BB3103, the ADAM10 (a desintegrin and metalloprotease) inhibitor that totally blocks the formation of sSortilin/NTSR3 in HT29 cells [6]. Targeting MMPs with drugs with a broad spectrum has been already tested in several cancer types, and although some interesting results have been obtained in terms of the reduction of cancer proliferation, the clinical tests have been canceled in phase I or in phase III for numerous inhibitors. MMPs are still promising targets with the further development of highly selective inhibitors for specific MMPs involved in each type of cancer (for review, see [70]).
